# Exploration of effective therapeutic approaches for vascular rupture complicated by acute vascular occlusion during middle cerebral artery balloon angioplasty

**DOI:** 10.1097/MD.0000000000046682

**Published:** 2025-12-12

**Authors:** Xudong Su, Dongjng Yao, Jianghua Yu, Yan Yan, Dandan Lin, Xiaoyun Liu

**Affiliations:** a Cerebrovascular Disease Department I, The First Hospital of Hebei Medical University, Shijiazhuang, China; b Brain Aging and Cognitive Neuroscience Laboratory of Hebei Province, Shijiazhuang, China; c Central Laboratory, The First Hospital of Hebei Medical University, Shijiazhuang, China; d Department of Neurology, The Second Hospital of Hebei Medical University, Shijiazhuang, China.

**Keywords:** complication, intraoperative hemorrhage, intraoperative thrombosis, middle cerebral artery, stent

## Abstract

**Rationale:**

Intraoperative hemorrhage and acute vascular occlusion during middle cerebral artery (MCA) balloon angioplasty constitute critical complications. The cooccurrence of these complications is rare and, if improperly managed, can lead to severe sequelae or become life-threatening.

**Patient concerns:**

A 64-year-old female patient presented with MCA stenosis. Imaging studies indicated watershed infarction in the corresponding blood supply area, along with vulnerable plaque and severe stenosis in the M1 segment of the right MCA.

**Diagnoses:**

Severe stenosis (>70%) of the right MCA with associated (watershed) cerebral infarction.

**Interventions:**

Following balloon angioplasty of the MCA, vascular rupture with hemorrhage occurred; immediate balloon occlusion achieved hemostasis. Subsequently, subtotal occlusion secondary to acute thrombus formation developed. The operator implanted a self-expanding stent with a planar coiled structure. This intervention successfully recanalized the occluded vessel without causing recurrent hemorrhage.

**Outcomes:**

Stent deployment successfully revascularized the right MCA stenosis, with intraoperative hemorrhage controlled and acute thrombotic occlusion resolved. The patient recovered without neurological deficits, achieving a modified Rankin Scale score of 0 at discharge.

**Lessons:**

In the course of balloon angioplasty for MCA stenosis, the occlusion of acute hemorrhage can be achieved through balloon dilation compression. Following the establishment of hemostasis, the treatment of acute vascular occlusion can be facilitated by the implantation of a “planar coiled structure” self-expanding stent, thereby successfully achieving recanalization of the MCA without inducing local vascular rehemorrhage.

## 1. Introduction

Balloon angioplasty and stent treatment for severe middle cerebral artery (MCA) stenosis is a common clinical treatment strategy. Intraoperative hemorrhage and acute vascular occlusion are both severe complications, although concurrent occurrence of the 2 is relatively rare. Improper management may lead to severe residual sequelae or even be life-threatening. Common hemostatic methods include balloon occlusion for hemostasis, neutralization of intraoperative heparin, and so forth.In severe hemorrhage, MCA occlusion may be necessary for hemostasis. Intraoperative thrombotic occlusion is a complex complication challenging to manage. To prevent recurrent hemorrhage, some clinicians choose to maintain vascular occlusion, but this risks severe postoperative deficits. Herein, we report the first case successfully recanalizing an occluded MCA by deploying a self-expanding stent with planar coil configuration without causing recurrent hemorrhage. A comprehensive literature search revealed no similar cases.

## 2. Case presentation

**Figure 1.**
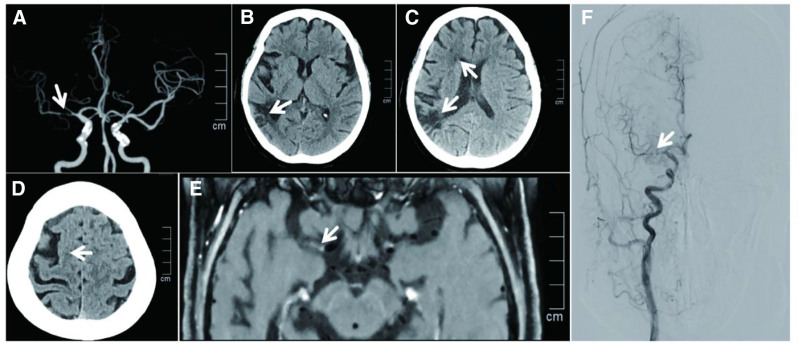
Preoperative CT, DSA, and high-resolution MRI findings. (A) CTA reveals severe stenosis in the right middle cerebral artery; (B) Right posterior watershed cerebral infarction; (C) Right anterior and posterior watershed infarction; (D) Right cerebral atrophy; (E) High-resolution MRI: shows diffuse severe stenosis of the right M1 segment with wall enhancement, indicative of an unstable plaque; (F) Preoperative DSA demonstrates severe stenosis in the M1 segment of the right middle cerebral artery (video0000BF05). CT = computed tomography, CTA = cranial computed tomography, DSA = digital subtraction angiography, MRI = magnetic resonance imaging.

**Figure 2.**
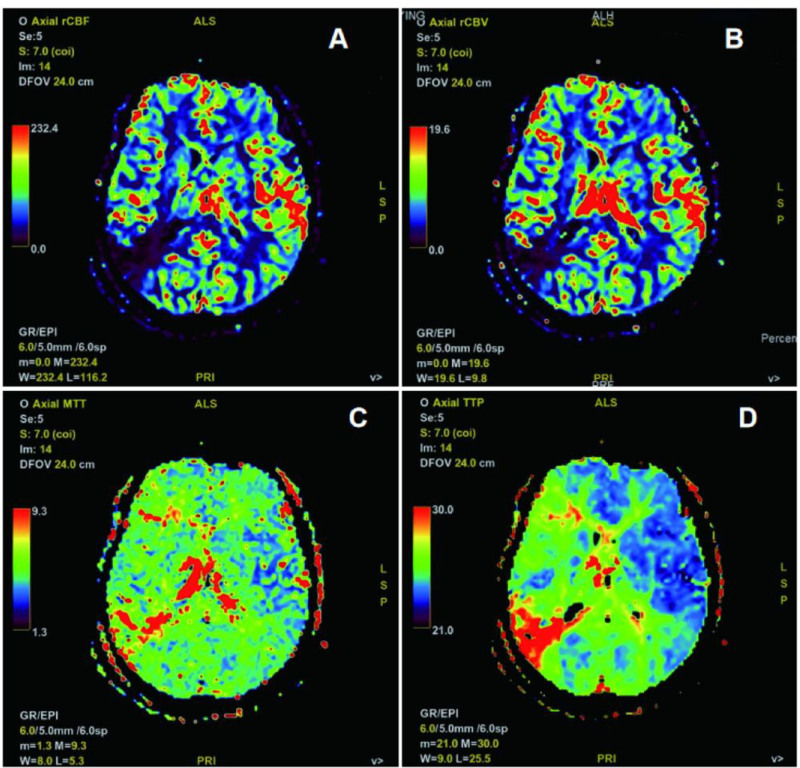
PWI demonstrates hypoperfusion in the territory of the right middle cerebral artery. (A) CBF is decreased; (B) CBV is reduced; (C) MTT is prolonged; (D) TTP is delayed. CBF = cerebral blood flow, CBV = cerebral blood volume, MTT = mean transit time, PWI = perfusion-weighted imaging, TTP = time to peak.

A 64-year-old male patient was admitted to the hospital with a diagnosis of watershed infarction in the blood supply area related to severe MCA stenosis. Cranial computed tomography of the cranium and cervical region was conducted upon admission, which revealed severe stenosis of the right MCA (Fig. 1A). Computed tomography (CT) revealed multiple infarcts in the right MCA blood supply area, with visible watershed infarction complicated by brain atrophy (Fig. 1B–D). The patient exhibited persistent headaches, with no documented history of hypertension or diabetes. Diagnosis: The patient exhibited severe stenosis of the right MCA and cerebral infarction. High-resolution magnetic resonance imaging performed after admission demonstrated a vulnerable plaque causing severe stenosis in the M1 segment of the right middle cerebral artery, accompanied by irregular arterial wall enhancement in the same segment as illustrated in the figure legend (Fig. 1E). Digital subtraction angiography (DSA) revealed diffuse severe stenosis of the M1 segment of the right MCA, accompanied by disuse atrophy of the distal vessels (Fig. 1F, video0000BF05). Perfusion-weighted imaging revealed lower cerebral blood volume and cerebral blood flow in the blood supply area of the right MCA compared with the contralateral side, along with prolonged mean transit time and time to peak, suggesting reduced blood perfusion (Table 1 and Fig. 2).Cranial MRI of the patient demonstrated multiple infarcts (including watershed infarcts) in the right middle cerebral artery territory, with marked atrophy of the brain tissue in the corresponding region, while physical examination revealed impaired higher cognitive function with a preoperative modified Rankin Scale (mRS) score of 1. A physical examination was conducted, which revealed an impairment of higher cognitive function. DSA confirmed diffuse severe stenosis of the M1 segment of the right MCA; perfusion-weighted imaging showed hypoperfusion in the corresponding blood supply area; and revealed unstable plaque accompanied by severe stenosis. The indications for endovascular treatment were considered. Following a period of 5 days during which the patient received regular dual antiplatelet therapy (aspirin 100 mg once daily, clopidogrel hydrogen sulfate 75 mg once daily), balloon angioplasty and stent implantation of the right MCA were scheduled.

**Table 1:** The results of PWI.

Axial scan plane	L/R	CBF	CBV	MTT	TTP
Centrum semiovale level	R	64.63	6.12	5.81	26.44
	L	101.30	8.35	4.90	24.56
Corona radiata level	R	79.81	7.07	5.52	26.45
	L	136.00	10.84	5.06	24.17
Basal ganglia level	R	95.27	8.35	5.40	24.89
	L	196.50	14.82	4.59	23.54

CBF = cerebral blood flow, CBV = cerebral blood volume, MTT = mean transit time, TTP = time to peak, PWI = perfusion-weighted imaging, TTP = time to peak.

**Figure 3.**
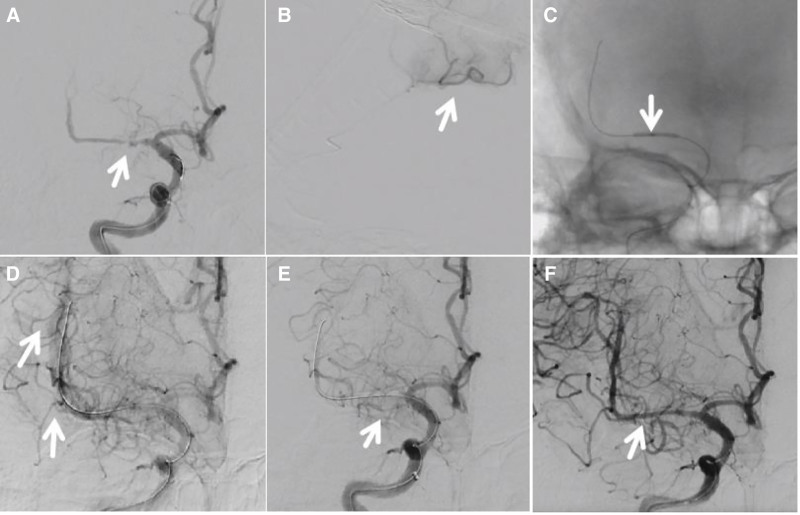
Intraoperative angiography results. (A) Preprocedural angiography reveals severe stenosis in the right middle cerebral artery with impaired distal perfusion (video0000C054); (B) After microcatheter advancement across the lesion, angiography confirms its position within the true lumen (video0000C055); (C) Balloon angioplasty (Gateway 1.5 × 15 mm, video0000C057); (D) Hemorrhagic rupture at the M1 segment (video0000C058); (E) M1 segment occlusion following successful hemostasis achieved by balloon tamponade (video0000C059); (F) Solitaire stent deployment results in good vascular reconstruction without evidence of rebleeding at the site of rupture (video0000C061).

The endovascular procedure was performed under general anesthesia via right radial access. A 6F distal access catheter was positioned in the C3 segment of the right internal carotid artery. An Echelon-10 microcatheter over a Synchro microguidewire was advanced through the right M1 stenosis; microcatheter angiography confirmed true lumen position (Fig. 3A, B, video0000C054/C055). A Gateway balloon (1.5 × 15 mm) was positioned across the stenotic site and inflated to 6 atmospheres for 1 minute (Fig. 3C, video0000C057). Angiography revealed contrast extravasation from a ruptured right M1 segment (Fig. 3D, video0000C058). The balloon was immediately reinserted and reinflated in the M1 segment to achieve hemostasis, simultaneous neutralization of heparin with protamine. After 3 minutes, balloon aspiration was performed; post-procedural angiography confirmed hemostasis. The balloon was withdrawn. During 10 minutes of observation, no recurrent hemorrhage occurred. However, acute thrombosis at the rupture site resulted in subtotal occlusion (Fig. 3E, video0000C059). A stent was deployed for vessel repair.

**Figure 4.**
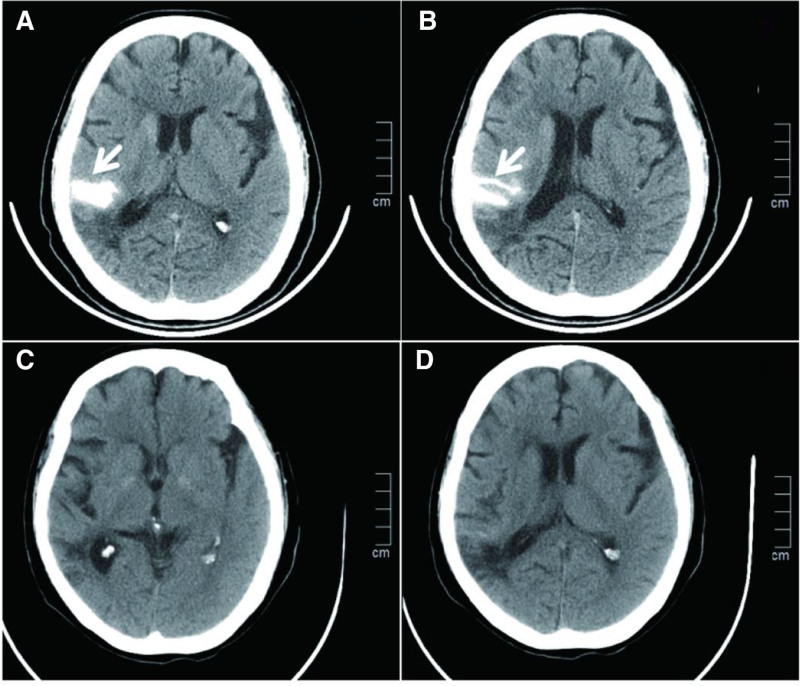
Postoperative cranial CT scans. (A, B) Scans show minimal subarachnoid hemorrhage; (C, D) Subsequent scan reveals complete resolution. CT = computed tomography.

A delivery microcatheter (Johnson & Johnson Plus) was passed through the lesion site. A self-expanding stent with a planar coil configuration (Solitaire AB 4.0 × 20 mm, ev3) was deployed through the microcatheter, covering the right M1 segment to the end of the internal carotid artery to release the stent. Angiography after stent implantation showed patency of the right MCA with no recurrent bleeding at the original site and adequate distal perfusion (Fig. 3F, video0000C061). During 50 minutes of observation, no recurrent hemorrhage occurred at the original site, with confirmed stent patency. The stent was then deployed. Postoperative cranial CT showed subarachnoid hemorrhage (Fig. 4A, B). After awakening from anesthesia, the patient complained only of headache. Neurological examination revealed no new deficits, with National Institutes of Health Stroke Scale and mRS scores of 0 and 1. By postoperative day 6, the headache had subsided. A follow-up head CT scan demonstrated that the hemorrhage was nearly absorbed, with no new cerebral infarction or neurological symptoms/signs (Fig. 4C, D). The patient was subsequently discharged. A 1-year telephone follow-up confirmed the absence of stroke recurrence, with preserved motor and speech function and a persistent mRS score of 1.

## 3. Discussion

^[1,2]^ Intracranial atherosclerotic stenosis (ICAS) is a major global risk factor for ischemic stroke, particularly prevalent in Asian populations.^[3]^ In China, 46.6% of symptomatic intracranial ischemic strokes involve ICAS, with MCA stenosis conferring high recurrent stroke risk.^[4]^ While medical therapy remains primary for ICAS,^[5]^ patients with severe stenosis and ischemic symptoms experience >20% annual stroke recurrence despite intensive medication.^[6]^ Consequently, percutaneous transluminal angioplasty with stenting has been investigated for severe ICAS.^[7]^ Musmar et al reviewed balloon-expandable stents versus balloon angioplasty alone for ICAS; prompt management of intraprocedural complications (e.g., thrombosis, wire entrapment) critically influences neurological prognosis.^[8]^ Zhu et al reported drug-coated balloon angioplasty for MCA stenosis. This approach may reduce intracranial foreign material retention and decrease postoperative antiplatelet dependence, potentially offering advantages in ICAS intervention.^[9]^ Periprocedural complications of MCA interventions—such as vascular rupture, acute thrombotic occlusion, and perforator injury—critically influence patient prognosis, as evidenced by clinical studies.^[10]^ Jiang et al identified perioperative hemorrhage in 3 out of 40 patients who received middle cerebral artery stenting, 1 of whom died.^[11]^ In a study of 196 patients implanted with middle cerebral artery stents, Wang et al reported 2 instances of intraoperative bleeding at the lesion site. Despite prompt treatment, both patients went on to develop new neurological symptoms.^[12]^ Okada et al also encountered 1 fatal hemorrhage among 47 patients treated with middle cerebral artery balloon angioplasty.^[13]^ Ueda et al noted 2 intraoperative hemorrhages in a cohort of 217 patients undergoing intracranial stenting or balloon angioplasty, though the clinical outcomes were not specified. Additionally,^[14]^ Wang et al published a case in which contrast extravasation around the stent occurred following middle cerebral artery stenting; the patient remained asymptomatic and achieved a favorable outcome. Taken together, these reports highlight that intraoperative vessel rupture carries significant risks of mortality or poor functional outcome. Conventional rescue strategies typically include neutralization of intraoperative heparin and balloon occlusion for hemostasis. In most published cases, patients sustained varying degrees of neurological impairment postoperatively, including some fatalities. Notably, the scenario described in our report—successful reconstruction of a vessel using a “flat-rolled structural stent” after iatrogenic occlusion following balloon hemostasis—has not, to our knowledge, been previously documented in the literature.

This report describes a patient with M1 segment MCA stenosis. Following balloon angioplasty, vascular rupture occurred. Hemostasis was achieved by balloon occlusion, but acute near-total occlusion developed in the M1 segment. A self-expanding stent with planar coil configuration was implanted, achieving vascular recanalization without recurrent hemorrhage at the rupture site. This approach prevented serious neurological complications. Literature on intraoperative hemorrhage and vascular occlusion during MCA interventions was reviewed.^[15]^ Marks et al (1999) reported a patient with ICAS who died intraoperatively from MCA rupture at the angioplasty site. Another patient developed thrombosis at the supraclinoid internal carotid artery angioplasty site 1 hour post-procedure. Intra-arterial tissue plasminogen activator achieved successful thrombolysis without neurological sequelae.^[16]^ Lee et al (2005) described a 58-year-old woman with diffuse severe stenosis of the right M1 segment. Balloon angioplasty caused vessel rupture, evidenced by contrast extravasation on angiography. Acute in-stent thrombosis occurred simultaneously. Hemostasis and recanalization were achieved by deploying a second overlapping stent at the rupture site with intra-arterial abciximab (10 mg). At 10-month follow-up, in-stent restenosis was observed without neurological deficits. This case demonstrates that stent deployment does not cause vessel rerupture.

This report details a patient with diffuse severe stenosis of the right MCA. This case report describes a patient with diffuse, severe stenosis of the right middle cerebral artery. The management options considered were endovascular intervention or open cerebral bypass surgery. Endovascular intervention is minimally invasive and allows for quicker recovery, whereas bypass surgery is more invasive, involves a prolonged recovery period, and extends the overall treatment timeline. Given the patient’s age, endovascular intervention was selected as the definitive treatment. The interventional procedure was considerably complicated by the patient’s unfavorable vascular anatomy and disuse atrophy in the local vascular bed. A Synchro microguidewire (Stryker) with soft tip was advanced with an Echelon microcatheter (ev3) through the stenosis, minimizing vascular injury risk. Microcatheter angiography confirmed true lumen position distal to the stenosis. Pre-dilation angiography showed no arterial dissection or vascular leakage. A small intracranial balloon (Gateway 1.5 × 15 mm) was selected for angioplasty with gradual inflation to minimize vascular injury risk. Post-dilation angiography revealed contrast extravasation from the M1 segment, indicating vascular rupture. Immediate heparin reversal and balloon occlusion achieved hemostasis within 3 minutes. After balloon withdrawal, subtotal M1 occlusion developed. The operator considered 2 options: procedure termination risking major cerebral infarction with hemiplegia, or continued angioplasty risking fatal rerupture. The operator noted the pre-rupture balloon diameter was smaller than the vessel lumen, reducing rupture risk from overdilation. A retrievable Solitaire AB stent with planar coil configuration was provisionally deployed in the M1 segment to restore flow while maintaining undeployed position. If rebleeding occurred, the stent could be retrieved and balloon reinflated immediately. Absence of hemorrhage would permit permanent stent deployment, preventing postoperative ischemic complications. After stent deployment, 50 minutes of observation confirmed no recurrent hemorrhage. The stent was then detached, completing the procedure. Postoperatively, the patient developed only headache with small-volume subarachnoid hemorrhage on imaging, without new neurological deficits. The patient recovered well and was discharged on day 6.

The patient’s preoperative laboratory studies included a C-reactive protein level of 3 mg/L (within normal range) and platelet aggregation rates of AA 11.6% and adenosine diphosphate 31.0%, consistent with adequate platelet inhibition under aspirin and clopidogrel therapy. Cranial computed tomography identified a noncalcified lesion at the affected site. High-resolution MRI demonstrated focal enhancement at the lesion, indicative of a vulnerable plaque. The stenosis appeared as a long-segment diffuse lesion rather than a focal narrowing, with an irregular luminal surface. DSA further revealed disuse atrophy involving both the stenotic segment and distal vasculature. During the procedure, the compromised local vasculature likely resulted in micro-dissection during microguidewire passage. Subsequent balloon dilation caused rupture and hemorrhage at the dissected site. Stent deployment successfully sealed the dissection without rebleeding, achieving perfect flow restoration in the target vessel. The patient recovered well and was discharged, with no stroke recurrence documented at 1-year follow-up. This case highlights that vulnerable plaque morphology, long-segment diffuse stenosis and disuse atrophy may represent risk factors for intraoperative vascular rupture and hemorrhage. These imaging characteristics could help identify patients at increased bleeding risk during intervention, though validation through larger cohort studies remains necessary.

Postoperative analysis indicated the hemorrhage likely originated from severe vascular lesions with impaired wall compliance and luminal degeneration. Microguidewire passage may have caused local dissection. Balloon inflation then ruptured this weakened site. Stent deployment apposed the dissection location, preventing rebleeding. Moreover, target vessel patency was restored. The patient recovered well postoperatively and had no stroke recurrence during 1-year follow-up.

## 4. Conclusion

Balloon angioplasty for MCA stenosis carries a risk of intraprocedural vascular rupture, a complication with high morbidity and mortality. After achieving hemostasis by balloon occlusion, subsequent thrombosis may prompt procedure termination. This decision risks new ischemic events in affected territories. This is the first reported case achieving MCA recanalization by combining balloon tamponade for hemostasis with deployment of a planar coil configuration self-expanding stent. This approach managed concurrent severe complications and stabilized the patient. Stent selection is critical: planar coil stents appose dissection flaps while exerting moderate radial force, reducing rerupture risk. This confirms that post-hemostasis thrombotic occlusion secondary to MCA angioplasty rupture can be treated with such stents. However, efficacy and safety require validation in large-scale studies.

## Author contributions

**Conceptualization:** Xudong Su, Dongjng Yao, Xiaoyun Liu.

**Data curation:** Xudong Su, Dongjng Yao, Yan Yan, Dandan Lin.

**Funding acquisition:** Xudong Su, Xiaoyun Liu.

**Investigation:** Xudong Su.

**Methodology:** Xudong Su.

**Project administration:** Xudong Su, Yan Yan, Dandan Lin.

**Writing – original draft:** Xudong Su, Dongjng Yao.

**Writing – review & editing:** Xudong Su, Jianghua Yu, Xiaoyun Liu.
